# A five-factor risk-stratification model for cancer-associated myositis in idiopathic inflammatory myopathies and stage-specific survival analysis: a multicentre Japanese MYKO cohort study

**DOI:** 10.3389/fimmu.2026.1914962

**Published:** 2026-08-11

**Authors:** Katsumasa Oe, Shogo Matsuda, Chifumi Akiyama, Mahiro Yamamoto, Takayasu Suzuka, Ran Nakashima, Hideaki Tsuji, Tsuneo Sasai, Yasuhiro Nohda, Tsuneyasu Yoshida, Yoichi Nakayama, Yuto Nakakubo, Atsubumi Ogawa, Kazuma Yoshida, Keisuke Hirobe, Yuki Aitani, Yudai Koshida, Hirofumi Miyake, Tohru Takeuchi, Takuya Kotani

**Affiliations:** 1 Department of Internal Medicine (IV), Division of Rheumatology, Osaka Medical and Pharmaceutical University, Osaka, Japan; 2 Department of Rheumatology and Clinical Immunology, Graduate School of Medicine, Kyoto University, Kyoto, Japan; 3 Department of General Internal Medicine, Tenri Hospital, Nara, Japan

**Keywords:** dermatomyositis, epidemiology, machine learning, mortality, neoplasia

## Abstract

**Introduction:**

Patients with idiopathic inflammatory myopathies (IIM) are at increased risk of malignancy, particularly around disease onset, but practical tools for risk stratification of cancer-associated myositis (CAM) remain limited. We aimed to characterise CAM in Japanese patients with IIM and develop a clinically applicable risk-stratification model.

**Methods:**

We conducted a multicentre retrospective study using the Japanese MYKO cohort, including patients diagnosed with IIM between 2001 and 2024. CAM was defined as malignancy diagnosed within ±3 years of IIM onset. Baseline demographic, clinical, laboratory, and autoantibody variables were compared between patients with and without CAM. Standardised incidence ratios (SIRs) were calculated using age-, sex-, and calendar year-specific cancer incidence rates from the Japanese general population. Random forest analysis and logistic regression were used to derive a CAM risk-stratification model. Overall survival and stage-specific survival were assessed using Kaplan–Meier analysis.

**Results:**

Among 364 patients with IIM, 42 (11.4%) had CAM. Most CAM cases occurred close to IIM onset, with 30 of 42 cases diagnosed within ±1 year. Compared with the general Japanese population, malignancy risk was increased within ±3 years of IIM onset (SIR 2.54, 95% CI 1.81–3.47) and was highest during the first year after onset (SIR 8.66, 95% CI 5.55–12.88). CAM was associated with older age at onset, male sex, smoking history, family history of malignancy, dermatomyositis subtype, dysphagia, elevated C-reactive protein (CRP) and anti-TIF1γ antibody positivity. Anti-TIF1γ positivity was the strongest predictor in random forest analysis. A five-factor model including anti-TIF1γ antibody positivity, elevated CRP, dermatomyositis subtype, older age at IIM onset, and family history of malignancy showed good discriminative performance (AUC 0.86; sensitivity 92.5%; specificity 65.1%). Five-year survival was lower in patients with CAM than in those without CAM (73.6% vs 94.6%) and was better in patients with early-stage than advanced-stage cancer (100% vs 44.0%).

**Conclusions:**

In this multicentre Japanese IIM cohort, malignancies in patients with CAM were diagnosed mainly around IIM onset. A machine learning-derived five-factor model identified patients at increased risk of CAM. An earlier-stage cancer at diagnosis was associated with better survival, highlighting the clinical importance of timely cancer detection.

## Introduction

Idiopathic inflammatory myopathies (IIM) are a heterogeneous group of rare systemic autoimmune diseases characterised by muscle inflammation and frequent extramuscular involvement of the skin, lungs, and joints (1). There are several subtypes of IIMs, including dermatomyositis (DM), polymyositis (PM), clinically amyopathic dermatomyositis (CADM), and anti-synthetase syndrome (ASyS) (1, 2).

Patients with IIM have an increased risk of malignancy, particularly within three years before or after the onset of IIM, a condition defined as cancer-associated myositis (CAM) (3, 4). In previous Japanese cohorts of patients with PM, DM, and CADM, malignancy was reported in approximately 15–25% of patients, with higher frequencies in patients with DM and CADM than in those with PM (5, 6).CAM has been associated with a poor prognosis, with reported 5-year survival rates ranging from 18% to 60% (5, 7–10). Because delayed cancer detection may worsen prognosis, timely and appropriate malignancy screening is essential for patients with IIM (3).

CAM also represents a model of malignancy-associated autoimmunity. Immune responses directed against tumour-associated antigens may overlap with autoimmune responses targeting muscle, skin, or other affected tissues (11). In particular, anti-transcription intermediary factor 1-gamma (TIF1γ) antibodies are strongly associated with cancer-associated dermatomyositis and provide a serological marker linking tumour immunity and systemic autoimmunity (11).

Several CAM risk factors have been identified mainly in previous non-Japanese cohorts and international meta-analyses, including older age at onset, high disease activity, dysphagia, DM subtype, necrotic skin lesions, and positivity for anti- TIF1γ or anti-nuclear matrix protein 2 antibodies (3, 12–15). The International Myositis Assessment and Clinical Studies Group (IMACS) has proposed a practical framework for IIM-associated cancer risk stratification and recommends regular malignancy screening for 3 years after IIM onset in high-risk patients (3). Previous machine-learning work has further identified cancer-associated clinical subtypes among anti-TIF1γ-positive myositis patients (16).

However, several clinically relevant gaps remain. Most established risk factors have been derived from studies conducted in Europe and the United States, and the clinical characteristics, predictors, and long-term outcomes of CAM in Japanese patients with IIM remain insufficiently studied (3, 5, 6, 8). Although international guidelines and recent prediction studies have advanced risk stratification for IIM-associated cancer, the optimal combination of clinical and laboratory factors for predicting CAM remains unclear. In addition, survival according to CAM status and cancer stage has not been sufficiently characterised. Previous Japanese studies of CAM were conducted at single centres and included relatively small CAM subgroups, ranging from 21 to 29 patients (5, 6, 8). Although these studies reported poor survival in patients with CAM, detailed survival analyses according to cancer stage were not provided. Therefore, stage-specific survival data in Japanese CAM populations remain limited.

To address these gaps, we conducted a multicentre retrospective study with long-term follow-up using the Japanese MYKO cohort. We aimed to identify the clinical characteristics and predictive factors of CAM, develop a clinically applicable risk-stratification model for CAM, and evaluate survival according to CAM status and cancer stage.

## Materials and methods

### Study design and patients

A multicentre, observational, retrospective study was conducted using the Japanese multicentre myositis registry, the Idiopathic Inflammatory Myopathy and Anti-synthetase Syndrome cohort of the Kansai Consortium (MYKO cohort) (17–19). We included patients from three participating institutions (Osaka Medical and Pharmaceutical University, Kyoto University, and Tenri Hospital) who were diagnosed with IIM between January 2001 and April 2024.

PM and DM were classified according to the Bohan and Peter criteria (20, 21), and CADM was classified according to Sontheimer’s criteria (22) and/or Gerami’s criteria (23), or the 2017 European Alliance of Associations for Rheumatology/American College of Rheumatology classification criteria (24). Patients who did not meet these criteria were subsequently evaluated for ASyS, which was defined by the Connors criteria, including anti-aminoacyl-tRNA synthetase (anti-ARS) antibody positivity with compatible clinical features such as myositis, interstitial lung diseases (ILD), fever, arthritis, Raynaud phenomenon, and mechanic’s hands (25). ILD was assessed using high-resolution computed tomography (HRCT), interpreted by pulmonary radiologists at each institution. Patients aged <18 years, those who did not fulfil any of the above classification criteria, and those with incomplete baseline data were excluded. The baseline variables were defined as those recorded at the time of IIM diagnosis. IIM onset was defined as the date of the first appearance of any core symptom suggestive of IIM as documented in the medical records. CAM was defined as IIM with a malignancy diagnosed within 3 years before or after IIM onset (3, 4); all other patients were classified as non-CAM. The timing of malignancy relative to IIM onset was categorised as pre- or post-onset; malignancies diagnosed within ±3 months of IIM onset were additionally described as a concurrent subset (9). For prognostic analyses, cancer stage was dichotomized as early stage (stage 0–II) and advanced stage (stage III–IV).

The study was conducted in accordance with the Declaration of Helsinki and its amendments (26), and was approved by the Faculty of Medicine Ethics Committee of Osaka Medical and Pharmaceutical University (approval no. 1529), Kyoto University (approval no. R1540), and Tenri Hospital (approval number: C23-11).

### Clinical and laboratory variables at baseline

In the MYKO cohort database, the following data were collected from the medical records: demographic characteristics including age at onset, sex, and disease duration; a family history of malignancy in second-degree relatives; clinical symptoms including fever, myalgia, muscle weakness, heliotrope rash, Gottron sign or papules, skin ulcer, joint pain, Raynaud phenomenon, and dysphagia; laboratory data including white blood cell count, albumin, C-reactive protein (CRP), creatine kinase, lactate dehydrogenase, ferritin, Krebs von den Lungen-6; myositis-specific antibody status; and the treatment details. Autoantibodies, including anti-ARS, anti-Jo-1, anti- melanoma differentiation-associated gene 5 (MDA5), anti-Mi-2, anti-TIF1γ, and anti-SS-A antibodies, were measured at each institution using clinically available assays, including enzyme-linked immunosorbent assay, chemiluminescent enzyme immunoassay, double immunodiffusion (Ouchterlony method), and/or immunoprecipitation assays.

### Treatments

All enrolled patients received glucocorticoids, including prednisolone. Additional treatments, including methylprednisolone pulse therapy, intravenous cyclophosphamide pulse therapy, cyclosporine, tacrolimus, azathioprine, mycophenolate mofetil, methotrexate, plasma exchange, and intravenous immunoglobulin were administered according to disease severity at the treating physician’s discretion.

### Statistical analysis

Patient characteristics are presented as medians (interquartile ranges) for continuous variables and as numbers (percentages) for categorical variables. The Mann–Whitney U test was used to compare continuous variables between the CAM and non-CAM groups, and Fisher’s exact test was used to compare categorical variables, as appropriate. Complete-case analysis was performed, and no imputation was used for missing data. Patients with incomplete baseline data required for the main analyses were excluded. The standardised incidence ratio (SIR) was calculated as the ratio of observed to expected cancers, with expected numbers derived by applying age-, sex-, and calendar year–specific cancer incidence rates from the National Cancer Center, Japan (MCIJ/Cancer Information Service) and e-Stat to accumulated person-years (27–29). As population incidence data were available until 2023, the follow-up for SIR estimation was censored on 31 December 2023. To ensure comparability with population-based incidence statistics that primarily report invasive cancers, *in situ* cancers were excluded from SIR analyses. Stratified analyses were performed according to sex, timing of malignancy relative to IIM onset, post-onset time bands, and cancer stage, where appropriate. No formal interaction analyses were performed.

To identify clinical, laboratory, and autoantibody predictors of CAM, we performed a random forest analysis (30) using the R package randomForest (version 4.7-1.2, http://cran.r-project.org/web/packages/randomForest/) as previously described (31, 32). Variable importance was assessed using the mean decrease in accuracy. Receiver operating characteristic (ROC) curve analyses were performed to determine the optimal cut-off value for predicting CAM. Given the limited number of outcome events, multivariable logistic regression was then performed using the top five predictors selected by the random forest to avoid overfitting. Continuous predictors were dichotomised using ROC-derived cutoffs, where applicable. The model discrimination was evaluated using the area under the ROC curve. Overall survival was assessed from IIM onset until death or the last confirmed follow-up using the Kaplan–Meier method, and patients who were alive at the last follow-up were censored. Differences between groups were evaluated using the log-rank test. No formal sensitivity analyses were performed. Statistical significance was set at *P* < 0.05. Statistical analyses were performed using R software (ver. 4.5.2), and JMP (version 18.0; SAS Institute Inc., Cary, NC, USA).

## Results

### Patient selection and cohort characteristics

A total of 487 patients clinically diagnosed with IIM were registered in the MYKO cohort between January 2001 and April 2024 (**Supplementary Figure 1**). Of these, 123 were excluded: 90 due to missing baseline data, 20 because they did not fulfil any diagnostic criteria for IIM as described in the Methods, and 13 with juvenile dermatomyositis. Therefore, 364 patients were included in the final analysis. The median patient age was 57 years and 28% of the patients were male. The IIM subtypes were DM in 129 (35%) patients, PM in 91 (25%), CADM in 115 (32%), and ASyS in 29 (8.0%). Anti-ARS antibodies were positive in 152 patients (42%) and anti-MDA5 antibodies in 94 (26%), anti-Mi-2 antibodies in 15 (4.1%), anti-TIF1γ antibodies in 24 (6.6%). The baseline characteristics are summarised in **Supplementary Table 1**.

### Malignancies in patients with IIM

**Figure 1:**
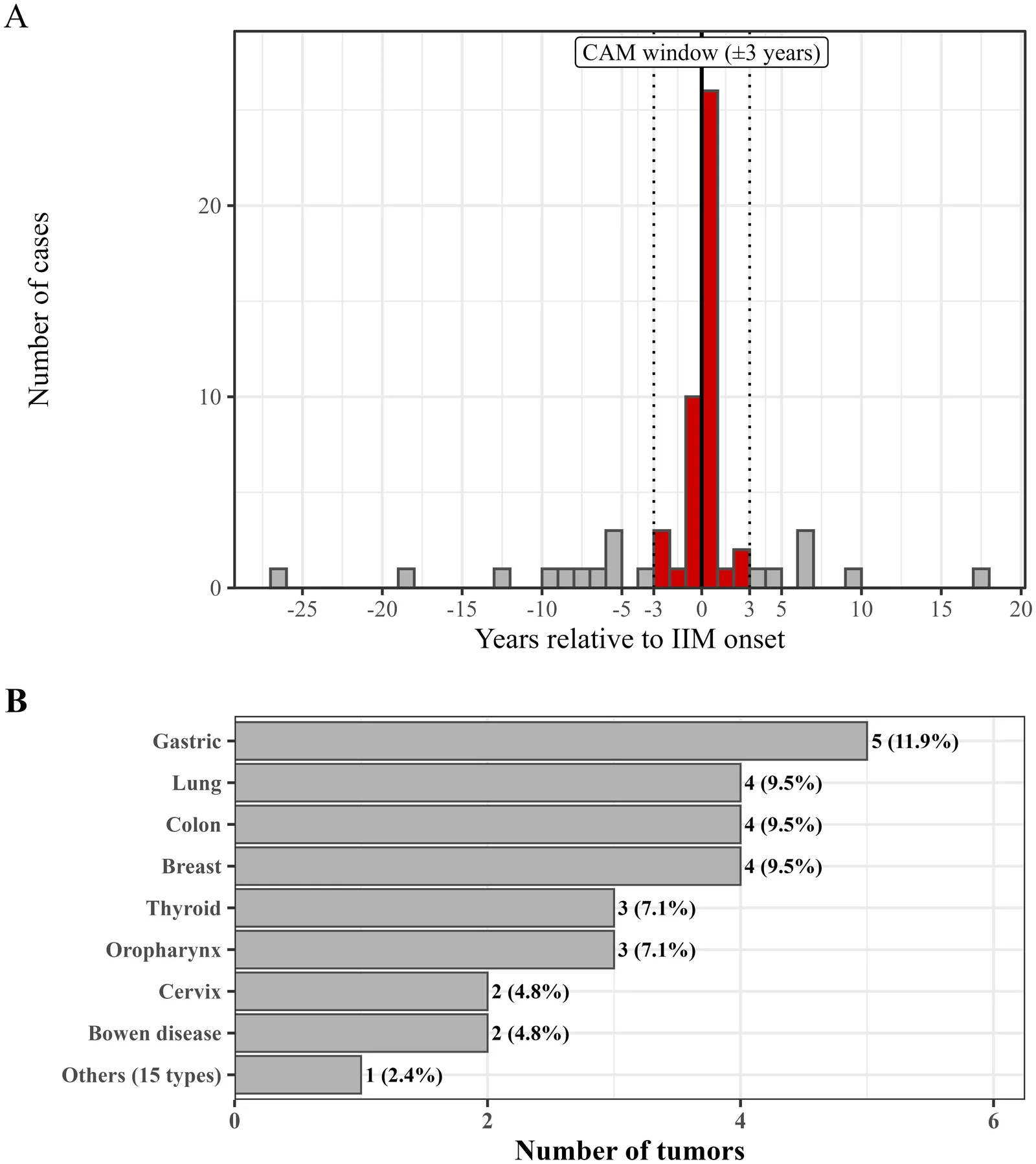
Timing and types of malignancy in idiopathic inflammatory myopathies. **(A)** Histogram showing timing of malignancy diagnosis relative to IIM onset (year 0). The CAM window (± 3 years) is indicated by dashed vertical lines; malignancies diagnosed within this window are highlighted in red, and those outside the window are shown in grey. Timing was known for 61 malignancies and unknown for 5. **(B)** Distribution of tumor types among patients with cancer-associated myositis (CAM; n=42); numbers indicate n (%). “Others” includes tumor types with a single case each. IIM, idiopathic inflammatory myopathies; CAM, cancer-associated myositis.

**Figure 2:**
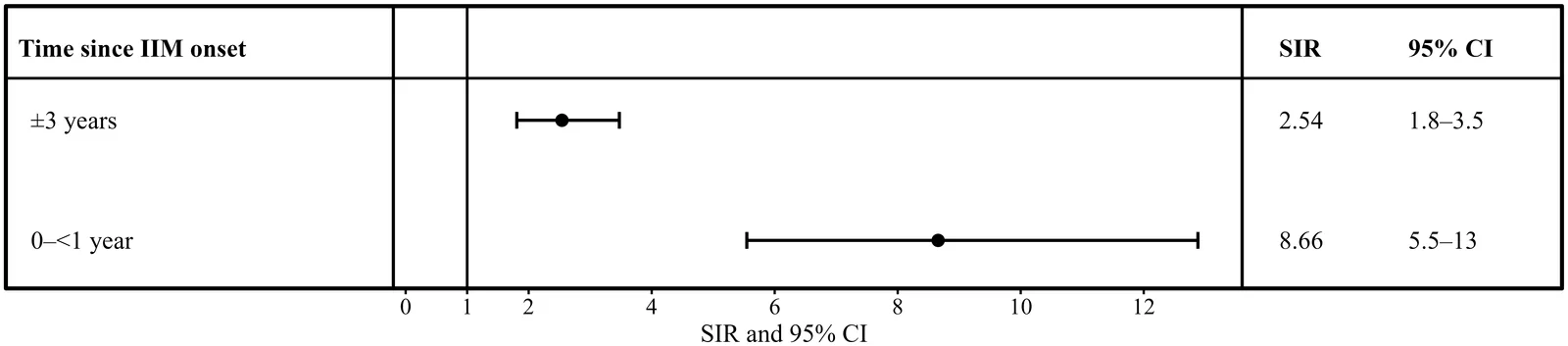
Standardized incidence ratios for malignancy in idiopathic inflammatory myopathies. Forest plot showing SIRs with 95% confidence intervals for malignancy diagnosed within ±3 years of IIM onset and within the first year after IIM onset. Expected numbers were derived from age-, sex-, and calendar year–specific cancer incidence rates in the Japanese general population. IIM, idiopathic inflammatory myopathies; SIR, standardized incidence ratio; CI, confidence interval.

Overall, 65 patients (17.9%) were diagnosed with malignancy during the observation period (**Figure 1A**). Of these, 42 (64.6%) were diagnosed within 3 years before or after IIM onset and were classified as having CAM. Among patients with CAM, malignancy was diagnosed pre-onset in 16 patients and post-onset in 26 patients; including 11 patients diagnosed concurrently (± 3 months from IIM onset). Overall, 30 malignancies were diagnosed within ±1 year of IIM onset. At the onset of IIM, systematic cancer screening was performed in a substantial proportion of patients, including computed tomography (CT) in 97.6%, upper gastrointestinal endoscopy in 69.0%, lower gastrointestinal endoscopy in 42.9%, gynaecological examinations in 28.6%, and breast imaging in 28.6%. The distribution of malignancy types is shown in **Figure 1B**. Among the patients with CAM (n = 42), gastric cancer was the most common (5 cases, 11.9%), followed by lung, colon, and breast cancers (4 cases each, 9.5%). Among the male patients with CAM (n = 18), lung cancer was the most frequent (4 cases, 22.2%), followed by gastric and colon cancers (2 cases each, 11.1%). Among female patients with CAM (n = 24), breast cancer (4 cases, 16.7%) and gastric cancer (3 cases, 12.5%) were predominant (**Supplementary Table 2**). Compared with the general Japanese population, the incidence of malignancy within ±3 years of IIM onset was significantly increased (SIR 2.54, 95% confidence interval [CI] 1.81–3.47). Post-onset stratification suggested that the risk was the highest during the first year (SIR 8.66, 95% CI 5.55–12.88) (**Figure 2**).

Among patients with CAM, 85.6% (36/42) received anticancer therapy. Anticancer treatment modalities included surgery in 71.4% (30 patients), chemotherapy in 35.7% (15 patients), and radiotherapy in 9.5% (4 patients).

### Comparison of clinical characteristics between CAM and non-CAM

Clinical characteristics were compared between patients with and without CAM (**Table 1**). Patients with CAM were significantly older (*P* < 0.001) and more frequently male (*P* = 0.027) compared with those without CAM. The CAM group more frequently had a history of smoking (*P* = 0.006), family history of malignancy (*P* = 0.006), dermatomyositis (*P* = 0.017), and dysphagia (*P* = 0.001). Serum CRP levels were significantly higher (*P* = 0.012), and anti-TIF1γ antibody positivity was more frequent in the CAM group (*P* < 0.001). The treatment patterns were largely comparable between groups. The initial prednisolone dose and use of intravenous cyclophosphamide and calcineurin inhibitors did not differ significantly. Intravenous immunoglobulin was administered more frequently in the CAM group (*P* = 0.004).

**Table 1:** Characteristics of patients with and without cancer-associated myositis.

Characteristics	N	non-CAM N = 322	CAM N = 42	P value
Initial characteristics				
Age at IIM onset, years	361	56 (46, 65)	68 (59, 77)	<0.001***
Male, n (%)	364	83 (26%)	18 (43%)	0.027*
Smoking history, n (%)	364	110 (34%)	25 (60%)	0.006**
Family history of malignancy†, n (%)	364	95 (30%)	22 (52%)	0.006**
Interstitial lung disease, n (%)	363	246 (77%)	26 (62%)	0.056
Dermatomyositis, n (%)	364	107 (33%)	22 (52%)	0.017*
Clinically amyopathic DM, n (%)	364	105 (33%)	10 (24%)	0.3
Polymyositis, n (%)	364	83 (26%)	8 (19%)	0.4
Anti-synthetase syndrome, n (%)	364	27 (8.4%)	2 (4.8%)	0.6
Clinical symptoms				
Fever, n (%)	340	59 (20%)	7 (18%)	0.8
Myalgia, n (%)	348	117 (38%)	16 (39%)	>0.9
Muscle weakness, n (%)	355	144 (46%)	23 (55%)	0.3
Heliotrope rash, n (%)	353	66 (21%)	13 (31%)	0.2
Gottron’s sign, n (%)	354	168 (54%)	26 (62%)	0.4
Skin ulcer, n (%)	330	15 (5.2%)	2 (5.0%)	>0.9
Joint pain, n (%)	345	110 (36%)	10 (24%)	0.2
Raynaud phenomenon, n (%)	332	43 (15%)	5 (13%)	0.8
Dysphagia, n (%)	364	30 (9.3%)	12 (29%)	0.001**
Initial laboratory features				
WBC,/μL	363	6,160 (4,790, 8,350)	6,500 (5,350, 8,970)	0.2
Albumin, g/dL	357	3.40 (3.00, 3.90)	3.25 (2.70, 3.50)	0.038*
CRP, mg/dL	347	0.30 (0.10, 1.20)	0.55 (0.20, 2.10)	0.012*
CK, U/L	364	267 (97, 1,046)	318 (84, 1,206)	0.6
LDH, U/L	361	340 (235, 495)	358 (308, 529)	0.088
Ferritin, ng/mL	248	237 (101, 624)	365 (140, 829)	0.11
KL-6, U/mL	293	646 (376, 1,063)	645 (466, 1,273)	0.5
Autoantibodies				
Anti-ARS antibody positive, n (%)	364	138 (43%)	14 (33%)	0.3
Anti-Jo-1 antibody positive, n (%)	364	49 (15%)	9 (21%)	0.4
Anti-MDA5 antibody positive, n (%)	364	87 (27%)	7 (17%)	0.2
Anti-Mi-2 antibody positive, n (%)	364	13 (4.0%)	2 (4.8%)	0.7
Anti-TIF1γ antibody positive, n (%)	364	10 (3.1%)	14 (33%)	<0.001***
Anti-SS-A antibody positive, n (%)	280	48 (19%)	3 (9.1%)	0.2
Treatment				
Initial prednisolone dose, mg/day	351	50 (40, 60)	55 (45, 60)	0.13
Methylprednisolone pulse therapy, n (%)	359	75 (24%)	11 (26%)	0.7
Intravenous cyclophosphamide, n (%)	359	134 (42%)	14 (33%)	0.3
Cyclosporine, n (%)	360	74 (23%)	9 (21%)	>0.9
Tacrolimus, n (%)	360	234 (74%)	28 (67%)	0.4
Azathioprine, n (%)	361	37 (12%)	7 (17%)	0.3
Mycophenolate mofetil, n (%)	362	59 (18%)	3 (7.1%)	0.081
Methotrexate, n (%)	359	40 (13%)	4 (9.5%)	0.8
Plasma exchange, n (%)	360	32 (10%)	3 (7.1%)	0.8
IVIg, n (%)	360	40 (13%)	13 (31%)	0.004**
Outcomes				
Follow-up duration from IIM onset, years	362	6.2 (3.0, 9.9)	3.7 (1.3, 6.6)	0.002**
Overall death, n (%)	364	27 (8.4%)	14 (33%)	<0.001***
Cancer-associated death, n (%)	364	3 (0.9%)	9 (21%)	<0.001***

Data are presented as median (IQR) or n (%).

N indicates the number of patients with available data; percentages are calculated using available cases.

Denominators vary owing to missing data.

*P* values were calculated using the Mann–Whitney U test for continuous variables and Fisher’s exact test for categorical variables.

Significance: *P < 0.05; **P < 0.01; ***P < 0.001.

†Second-degree relatives.

IIM, idiopathic inflammatory myopathies; CAM, cancer-associated myositis; DM, dermatomyositis; CADM, clinically amyopathic dermatomyositis; PM, polymyositis; ASyS, anti-synthetase syndrome; ILD, interstitial lung disease; WBC, white blood cell; CRP, C-reactive protein; CK, creatine kinase; LDH, lactate dehydrogenase; KL-6, Krebs von den Lungen-6; ARS, aminoacyl-tRNA synthetase; Jo-1, histidyl-tRNA synthetase; MDA5, melanoma differentiation-associated gene 5; Mi-2, nucleosome remodeling-deacetylase complex subunit; TIF1γ, transcription intermediary factor 1-gamma; SS-A, Sjögren’s-syndrome-related antigen A; IVIg, intravenous immunoglobulin; IQR, interquartile range.

### Random forest analysis for CAM-associated variable selection

**Figure 3:**
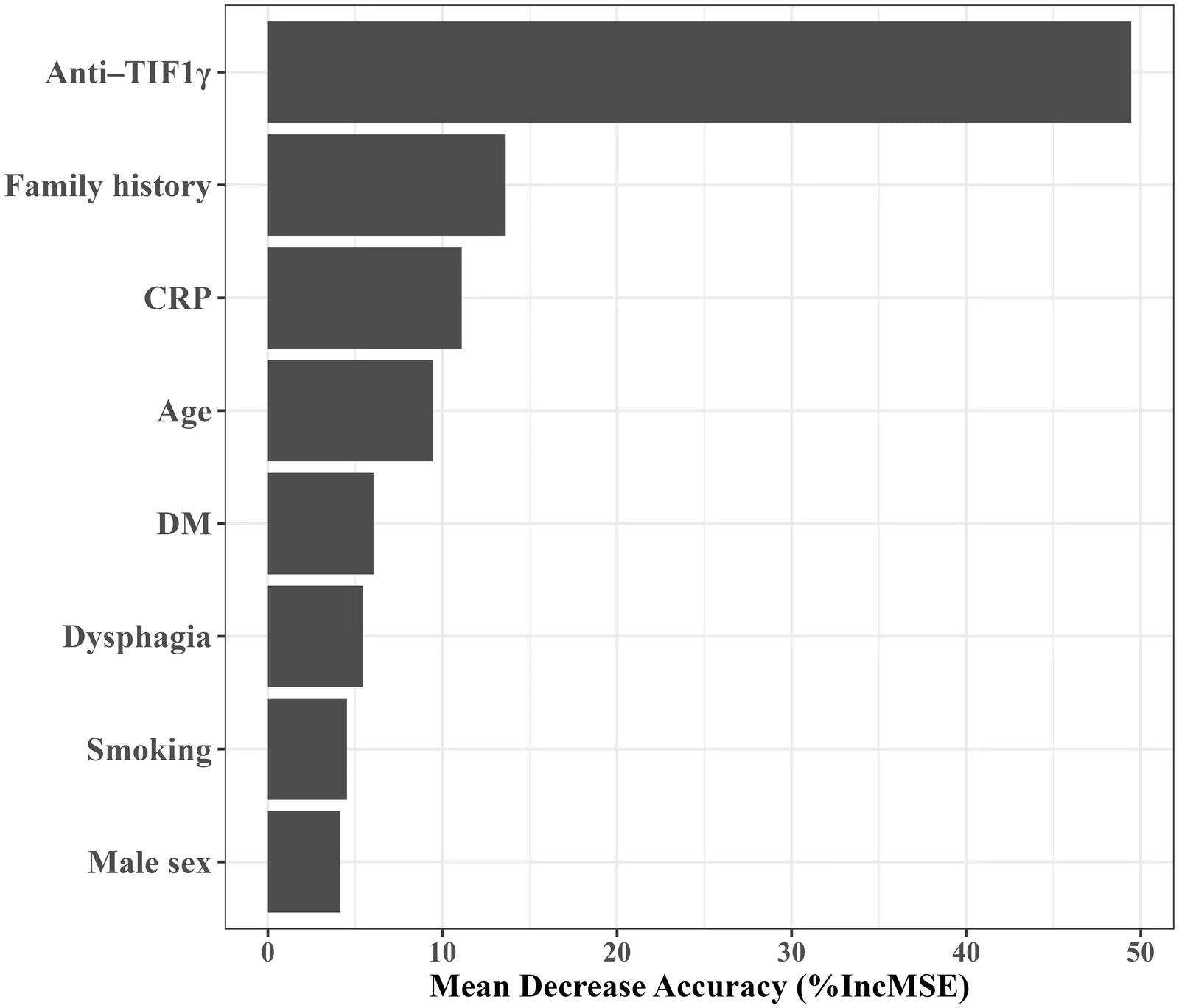
Variable importance for predicting cancer-associated myositis. Bar plot of variable importance from the random forest model, expressed as mean decrease in accuracy. Higher values indicate greater contribution to classification performance. Family history denotes family history of malignancy (see Methods for definition). CAM, cancer-associated myositis; CRP, C-reactive protein; DM, dermatomyositis.

To identify optimal predictors of CAM, we performed random forest analysis including variables that were significant in the univariate analysis: age, sex, DM subtype, dysphagia, smoking history, serum CRP levels, anti-TIF1γ antibody status, and family history of malignancy. In the variable-importance analysis, anti-TIF1γ positivity was the strongest predictor (mean decrease accuracy 49.5), followed by family history of malignancy (13.6), CRP level (11.1), age (9.4), and DM subtype (6.1) (**Figure 3**).

### Five-factor model for CAM risk stratification

Subsequently, we developed a logistic regression model using the top five predictors ranked by mean decrease accuracy: anti-TIF1γ antibody positivity, family history of malignancy, CRP level, age, and DM subtype. ROC curve analysis identified optimal cut-off values of age ≥ 69 years and CRP ≥ 0.17 mg/dL (**Supplementary Figure 2**). As shown in **Table 2**, the five-factor model combining all these predictors demonstrated the best discriminative performance (AUC 0.86), with a sensitivity of 92.5% and specificity of 65.1%. All combinations of candidate predictors were evaluated using logistic regression and their performance metrics are summarised in **Supplementary Table 3**. To facilitate clinical interpretation of the five-factor model, we summarized observed CAM frequency and model-predicted probability according to the number of final-model risk factors present (**Supplementary Table 4**, **Supplementary Figure 3**). The observed CAM frequency increased from 0.0% in patients with no risk factors to 88.9% in those with four or more risk factors.

**Table 2:** Top 10 logistic regression models for prediction of cancer-associated myositis.

Predictors	AUC	Sensitivity	Specificity	AIC
TIF1γ + CRP ≥ 0.17 mg/dL + DM + Age ≥ 69 years + Family history	0.857	92.5	65.1	187.3
TIF1γ + CRP ≥ 0.17 mg/dL + Age ≥ 69 years + Family history	0.851	92.5	65.5	186.2
TIF1γ + DM + Age ≥ 69 years + Family history	0.839	77.5	75.3	199.8
TIF1γ + Age ≥ 69 years + Family history	0.831	65.0	82.9	199.4
TIF1γ + CRP ≥ 0.17 mg/dL + DM + Family history	0.809	80.0	73.7	199.6
TIF1γ + CRP ≥ 0.17 mg/dL + Family history	0.807	80.0	73.7	197.8
CRP ≥ 0.17 mg/dL + DM + Age ≥ 69 years + Family history	0.801	75.0	71.1	215.6
TIF1γ + CRP ≥ 0.17 mg/dL + DM + Age ≥ 69 years	0.794	57.5	87.5	195.0
TIF1γ + CRP ≥ 0.17 mg/dL + Age ≥ 69 years	0.789	57.5	89.1	193.9
TIF1γ + DM + Age ≥ 69 years	0.782	65.0	82.9	204.4

Models were ranked by AUC, then AIC.

AUC was calculated from ROC curves of predicted probabilities from each logistic regression model.

Sensitivity and specificity are reported as percentages and were calculated at the optimal probability threshold based on the Youden index.

AUC, area under the receiver operating characteristic curve; AIC, Akaike information criterion; CRP, C-reactive protein; DM, dermatomyositis.

### Survival in patients with CAM

**Figure 4:**
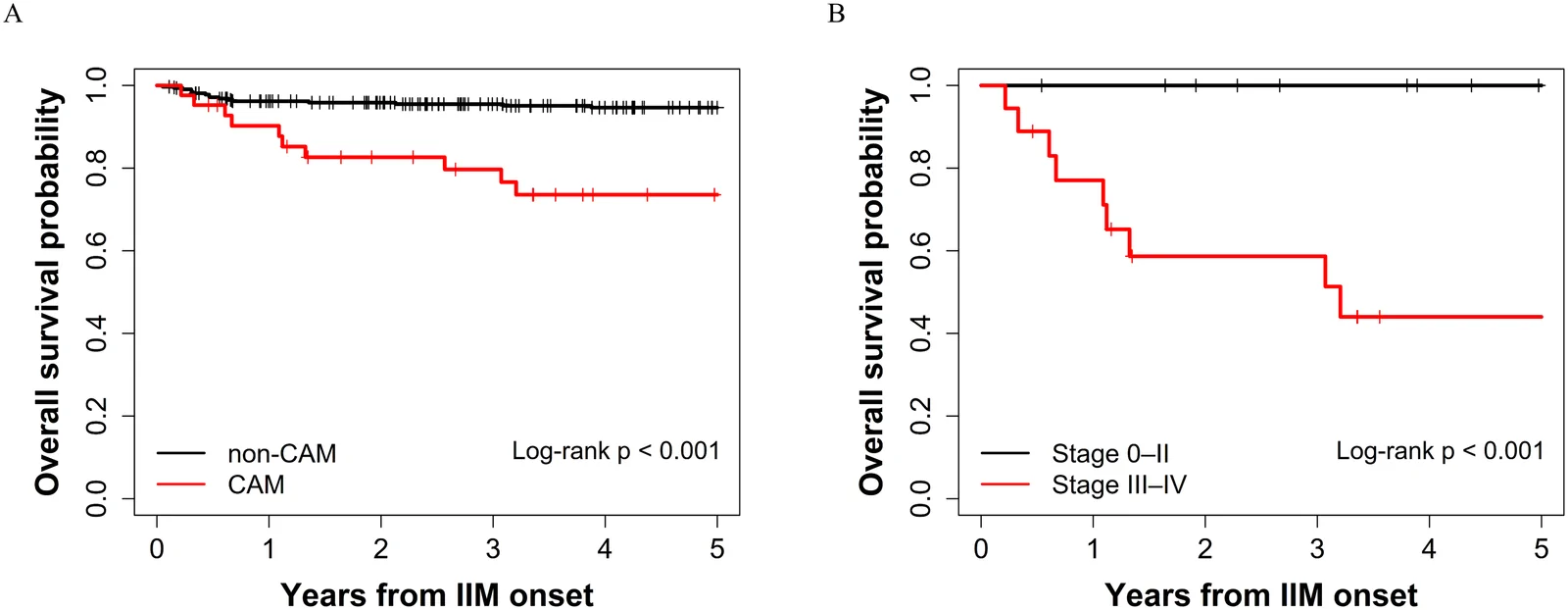
Overall survival according to cancer-associated myositis status and cancer stage. **(A)** Kaplan–Meier curves comparing overall survival between patients with cancer-associated myositis (CAM) and those without CAM (non-CAM) from the time of IIM onset. **(B)** Kaplan–Meier curves for overall survival among CAM patients stratified by cancer stage (stage 0–II vs stage III–IV) from the time of IIM onset. Tick marks indicate censored observations. P values were calculated using the log-rank test. CAM, cancer-associated myositis; IIM, idiopathic inflammatory myopathies.

During follow-up, 14 patients (33.3%) died at a median of 1.9 (0.8–5.8) years from IIM onset; nine deaths (64.3%) were attributable to malignancy. Overall, 14 of the 42 patients with CAM and 27 of the 322 patients without CAM died during the observation period. Kaplan–Meier analysis demonstrated a significantly poorer overall survival in the CAM group than in the non-CAM group (log-rank *P* < 0.0001), with estimated 5-year survival rates of 73.6% and 94.6%, respectively (**Figure 4A***).*

Among the patients with CAM, the cancer stage at diagnosis was stage 0 (carcinoma *in situ*) in 9.5%, stage I in 33.3%, stage II in 9.5%, stage III in 16.7%, and stage IV in 26.2%; the cancer stage was unknown in 4.8%. Kaplan–Meier analysis demonstrated significantly poorer overall survival in patients with advanced-stage cancer (stages III–IV) than in those with early-stage cancer (stages 0–II) (log-rank *P* < 0.001) (**Figure 4B**). The estimated 5-year survival rate was 100% in the early-stage group, with no deaths observed, and 44.0% in the advanced-stage group.

## Discussion

In this multicentre Japanese IIM cohort, we characterised the clinical and prognostic features of CAM and developed a five-factor model for CAM risk stratification based on routinely available variables. Among 364 patients with IIM, 42 patients (11.4%) were classified as having CAM, and most cases were diagnosed close to IIM onset, with 30 of 42 CAM cases occurring within ±1 year. Compared with the general Japanese population, the incidence of malignancy was increased, particularly during the first year after IIM onset. We developed a five-factor model incorporating anti-TIF1γ antibody positivity, elevated CRP, dermatomyositis subtype, older age at IIM onset, and family history of malignancy, which showed good discriminative performance using routinely available clinical variables. Importantly, survival was markedly better in patients with early-stage cancer than in those with advanced-stage disease. Together, these findings support a risk-stratified approach to malignancy surveillance in IIM and provide a basis for future external validation of a clinically applicable CAM prediction model. The prevalence of CAM in our cohort (11.4%) was consistent with previously reported rates in Asian and Western populations. Previous Japanese cohorts have reported malignancy in approximately 15–25% of patients with PM, DM, or CADM (5, 6), and studies from Taiwan, China, and Australia have similarly reported malignancy in approximately 10–25% of patients with IIM (33–35). These findings suggest that CAM represents a reproducible clinical phenotype across different populations.

In our CAM cohort, gastric cancer was the most common malignancy, followed by lung, colon, and breast cancers. Notably, the distribution differed by sex; lung cancer was the most frequent in men, whereas breast cancer was the most frequent in women. This pattern is consistent with that of Japanese and other East Asian cohorts reporting sex-specific differences in cancer types among patients with IIM (5, 34). In contrast, region-specific patterns, such as nasopharyngeal carcinoma, reported as a leading malignancy in Taiwanese cohorts (33), were not observed in our cohort. These findings suggest that the spectrum of cancers associated with IIM is influenced by geography and background cancer epidemiology. In clinical practice in Japan, it may be reasonable to consider a targeted cancer surveillance approach focusing on gastrointestinal malignancies in all patients, chest HRCT for lung cancer in men, and breast imaging in women.

Notably, most CAM cases were diagnosed around the time of IIM onset, with 30 of 42 patients (71.4%) diagnosed within ±1 year. This temporal pattern is consistent with prior reports (10, 36). Our SIR analyses showed that the overall SIR within ±3 years of IIM onset was increased compared with the age-adjusted general Japanese population, and that, among post-onset time bands, the SIR was the highest in the first year. These findings support comprehensive malignancy screening at IIM onset and careful follow-up during the subsequent three years, especially in patients with additional CAM-associated risk factors.

Furthermore, univariate analyses identified older age, male sex, DM subtype, anti-TIF1γ antibody positivity, a family history of malignancy, smoking history, dysphagia, and elevated serum CRP levels as factors associated with CAM. Among these factors, the associations of older age, male sex, DM subtype, anti-TIF1γ antibody positivity, a family history of malignancy, smoking, dysphagia with cancer risk in IIM were consistent with previous reports (12, 15). Additionally, we found that serum CRP levels were significantly higher in patients with CAM. Elevated serum CRP levels have been reported in various malignancies and reflect systemic inflammatory responses related to tumour burden, host immune activation, or cancer-associated inflammation (37). These findings suggest that systemic inflammatory activity, as reflected by CRP levels, may be associated with CAM.

Although several risk factors for CAM have been reported previously (3, 12–15), most studies were based on small sample sizes or single-centre cohorts, and few predictive models incorporating multiple risk factors have been established. In particular, evidence of machine learning–based prediction tools for CAM in the Japanese population remains limited. To the best of our knowledge, this is the first study to develop and internally evaluate a CAM risk prediction model using a machine learning approach in a multicentre Japanese IIM cohort. In the present study, random forest analysis identified anti-TIF1γ antibody positivity, family history of malignancy, CRP level, age at IIM onset, and DM subtype as the top five predictors of CAM (**Figure 3**). A model incorporating these five routinely assessable factors showed the best discriminative performance for CAM among the evaluated combinations. The IMACS provides a practical framework for risk-stratified malignancy screening in IIM (3). However, the optimal combination of predictors and their performance across different populations remains to be determined. Our multicentre Japanese cohort study demonstrated that the combination of these five factors allows early risk stratification for CAM. Furthermore, because all five factors are routinely assessable in clinical practice, this model may help identify patients who require more intensive malignancy surveillance at IIM diagnosis and during the subsequent three years.

Anti-TIF1γ antibody positivity was the strongest single predictor of CAM, but CAM risk was not fully captured by this marker alone. In the present study, anti-TIF1γ positivity alone showed high specificity but limited sensitivity (**Supplementary Table 3**), whereas the five-factor model improved overall discrimination. Previous clinicohistopathological data also support the heterogeneity of CAM. Hida et al. showed that anti-TIF1γ-positive CAM accounted for approximately half of CAM cases and was characterised by close temporal association with cancer detection, whereas anti-TIF1γ-negative CAM included diverse IIM subgroups, including necrotizing autoimmune myopathy (38). A prior machine-learning study refined cancer prediction among patients with anti-TIF1γ-positive myositis (16). Complementing this work, our study suggests that CAM risk assessment in the broader IIM population may benefit from integrating anti-TIF1γ status with additional clinical and inflammatory factors.

Previous studies have reported poor outcomes in CAM, with 5-year mortality ranging from approximately 25% to 60% (5, 7, 8). In contrast, our study demonstrated a relatively favourable prognosis with a 5-year survival rate of 73.6%, which was higher than that previously reported in Japanese cohorts (5, 8). One possible explanation is that the comprehensive malignancy screening performed around the onset of IIM in this cohort, including CT, gastrointestinal endoscopy, gynaecological examinations, and breast imaging, may have resulted in earlier cancer detection, which was associated with better survival in our cohort. Nevertheless, patients diagnosed with advanced-stage malignancy have markedly poorer survival rates than those diagnosed with early-stage disease. These findings emphasise the clinical importance of early cancer detection in high-risk IIM patients and highlight the need for biomarkers and refined risk-stratified screening strategies to improve the early recognition of malignancy.

This study had several limitations. First, because of the retrospective design and exclusion of patients with incomplete baseline data, selection bias may have occurred. Second, the cohort was derived from a Japanese population, and further studies are required to determine whether these findings can be generalised to other ethnicities and healthcare settings. Third, the selection of malignancy screening methods was left to the discretion of the treating physicians, which may have led to variability in the diagnostic approach and potential detection bias in the identification of CAM. Fourth, the CAM risk prediction model developed in this study has not yet undergone external validation; therefore, its performance in independent cohorts remains to be established. Fifth, anti-NXP2 antibody status was not systematically evaluated across participating centres and was therefore not included as a candidate variable in the present analysis.

Despite these limitations, this study had several strengths. We developed a CAM risk prediction model using a machine learning approach and evaluated it in a multicentre cohort of patients with IIM with long-term follow-up. This study included a relatively large, well-characterised cohort of 364 Japanese patients with IIM, with standardised baseline assessments and centralised data collection, which strengthened the internal validity of our findings.

## Conclusion

In this multicentre Japanese IIM cohort, malignancies in patients with CAM were diagnosed mainly around IIM onset. A machine learning-derived five-factor model identified patients at increased risk of CAM. An earlier-stage cancer at diagnosis was associated with better survival, highlighting the clinical importance of timely cancer detection.

## Data Availability

The data that support the findings of this study are not publicly available because they consist of individual-level clinical data and are subject to institutional and ethical restrictions. The data may be made available from the corresponding author upon reasonable request and with permission from the relevant institutions.

## Supplementary material

Supplementary Figure 1 Patient selection flow diagram. Flowchart of cohort inclusion and exclusions in the MYKO registry. Of 487 patients with clinically diagnosed idiopathic inflammatory myopathies (IIM), 123 were excluded (insufficient clinical data, n=90; did not meet diagnostic criteria, n=20; juvenile dermatomyositis, n=13), leaving 364 patients with available baseline data at diagnosis. The final cohort was classified as cancer-associated myositis (CAM; n=42) or non-CAM (n=322). IIM, idiopathic inflammatory myopathies; CAM, cancer-associated myositis.

Supplementary Figure 2 Receiver operating characteristic curves for prediction of cancer-associated myositis. ROC curves for age at IIM onset (A) and serum CRP (B) as predictors of CAM. The dashed line indicates no discrimination. Optimal cut-offs were age ≥69 years and CRP ≥0.17 mg/dL. CAM, cancer-associated myositis; CRP, C-reactive protein; IIM, idiopathic inflammatory myopathies; ROC, receiver operating characteristic.

Supplementary Figure 3 Observed and model-predicted probability of cancer-associated myositis according to the number of final-model risk factors present. Bars indicate the observed frequency of cancer-associated myositis (CAM), and points with error bars indicate the median and interquartile range of model-predicted CAM probability calculated using the final five-factor logistic regression model. Patients with four or five risk factors were combined into a ≥4 category because only one patient had all five risk factors.

Supplementary Table 1 Baseline characteristics of the overall idiopathic inflammatory myopathy cohort (N = 364). Data are presented as median (interquartile range) or n (%). Denominators vary owing to missing data.

Supplementary Table 2 Types of malignancy among patients with cancer-associated myositis. Data are presented as n (%). Percentages are calculated within each section (overall, male, and female). Each patient contributed one malignancy diagnosis within the cancer-associated myositis window.

Supplementary Table 3 All logistic regression model combinations for prediction of cancer-associated myositis. All 31 possible combinations of the candidate predictors were evaluated. Models were ranked by area under the receiver operating characteristic curve and then by Akaike information criterion. Sensitivity and specificity are presented as percentages and were calculated at the optimal probability threshold determined by the Youden index for each model. k denotes the number of predictors included in each model.

Supplementary Table 4 Observed and model-predicted probability of CAM according to the number of final-model risk factors present.
