# Supplementary material for: A five-factor risk-stratification model for cancer-associated myositis in idiopathic inflammatory myopathies and stage-specific survival analysis: a multicentre Japanese MYKO cohort study
Source: Front Immunol. 2026 Aug 11;17:1914962. doi: 10.3389/fimmu.2026.1914962 (PMC13504253; doi:10.3389/fimmu.2026.1914962)
Supplement: Supplementary file 1 [file Table1.docx]

**Supplementary Table S1. Baseline characteristics of the overall idiopathic inflammatory myopathies cohort.**

| **Characteristics** | **N = 364** |
| --- | --- |
| **Initial characteristics** | |
| Age at IIM onset, years | 57 (47, 66) |
| Male, n (%) | 101 (28%) |
| Smoking history, n (%) | 135 (37%) |
| Family history of malignancy†, n (%) | 117 (32%) |
| Interstitial lung disease, n (%) | 272 (75%) |
| Dermatomyositis, n (%) | 129 (35%) |
| Clinically amyopathic DM, n (%) | 115 (32%) |
| Polymyositis, n (%) | 91 (25%) |
| Anti-synthetase syndrome, n (%) | 29 (8.0%) |
| **Clinical symptoms** | |
| Fever, n (%) | 66 (19%) |
| Myalgia, n (%) | 133 (38%) |
| Muscle weakness, n (%) | 167 (47%) |
| Heliotrope rash, n (%) | 79 (22%) |
| Gottron's sign, n (%) | 194 (55%) |
| Skin ulcer, n (%) | 17 (5.2%) |
| Joint pain, n (%) | 120 (35%) |
| Raynaud phenomenon, n (%) | 48 (14%) |
| Dysphagia, n (%) | 42 (12%) |
| **Initial laboratory features** | |
| WBC, /μL | 6,220 (4,800, 8,450) |
| Albumin, g/dL | 3.40 (3.00, 3.90) |
| CRP, mg/dL | 0.30 (0.10, 1.30) |
| CK, U/L | 267 (94, 1,150) |
| LDH, U/L | 343 (246, 495) |
| Ferritin, ng/mL | 257 (107, 675) |
| KL-6, U/mL | 645 (386, 1,078) |
| **Autoantibodies** | |
| Anti-ARS antibody positive, n (%) | 152 (42%) |
| Anti-Jo-1 antibody positive, n (%) | 58 (16%) |
| Anti-MDA5 antibody positive, n (%) | 94 (26%) |
| Anti-Mi-2 antibody positive, n (%) | 15 (4.1%) |
| Anti-TIF1γ antibody positive, n (%) | 24 (6.6%) |
| Anti-SS-A antibody positive, n (%) | 51 (18%) |
| **Treatment** | |
| Initial prednisolone dose, mg/day | 50 (40, 60) |
| Methylprednisolone pulse therapy, n (%) | 86 (24%) |
| Intravenous cyclophosphamide, n (%) | 148 (41%) |
| Cyclosporine, n (%) | 83 (23%) |
| Tacrolimus, n (%) | 262 (73%) |
| Azathioprine, n (%) | 44 (12%) |
| Mycophenolate mofetil, n (%) | 62 (17%) |
| Methotrexate, n (%) | 44 (12%) |
| Plasma exchange, n (%) | 35 (9.7%) |
| IVIg, n (%) | 53 (15%) |
| **Outcomes** | |
| Follow-up duration from IIM onset, years | 5.8 (2.7, 9.1) |
| Overall death, n (%) | 41 (11%) |
| Cancer-associated death, n (%) | 12 (3.3%) |
| Data are presented as median (IQR) or n (%). | |
| Denominators vary owing to missing data. | |
| †Second-degree relatives. | |
| Abbreviations: IIM, idiopathic inflammatory myopathies; DM, dermatomyositis; CADM, clinically amyopathic dermatomyositis; PM, polymyositis; ASyS, anti-synthetase syndrome; ILD, interstitial lung disease; WBC, white blood cell; CRP, C-reactive protein; CK, creatine kinase; LDH, lactate dehydrogenase; KL-6, Krebs von den Lungen-6; ARS, aminoacyl-tRNA synthetase; Jo-1, histidyl-tRNA synthetase; MDA5, melanoma differentiation-associated gene 5; Mi-2, nucleosome remodeling-deacetylase complex subunit; TIF1γ, transcription intermediary factor 1-gamma; SS-A, Sjögren’s-syndrome-related antigen A; IVIg, intravenous immunoglobulin; IQR, interquartile range. | |

Legend:

Supplementary Table S1. Baseline characteristics of the overall idiopathic inflammatory myopathy cohort (N=364). Data are presented as median (interquartile range) or n (%). Denominators vary owing to missing data.
