# Supplementary material for: A five-factor risk-stratification model for cancer-associated myositis in idiopathic inflammatory myopathies and stage-specific survival analysis: a multicentre Japanese MYKO cohort study
Source: Front Immunol. 2026 Aug 11;17:1914962. doi: 10.3389/fimmu.2026.1914962 (PMC13504253; doi:10.3389/fimmu.2026.1914962)
Supplement: Supplementary file 2 [file Table2.docx]

**Supplementary Table S2. Types of malignancy among patients with cancer-associated myositis.**

| Tumor type | n (%) |
| --- | --- |
| **Overall (n=42)** | |
| Gastric | 5 (11.9%) |
| Breast | 4 (9.5%) |
| Colon | 4 (9.5%) |
| Lung | 4 (9.5%) |
| Oropharynx | 3 (7.1%) |
| Thyroid | 3 (7.1%) |
| Bowen disease | 2 (4.8%) |
| Cervix | 2 (4.8%) |
| Bladder | 1 (2.4%) |
| Duodenum | 1 (2.4%) |
| Extramammary Paget’s | 1 (2.4%) |
| Gallbladder | 1 (2.4%) |
| Gum | 1 (2.4%) |
| Hepatic | 1 (2.4%) |
| Lymphoma | 1 (2.4%) |
| Oral | 1 (2.4%) |
| Ovarian | 1 (2.4%) |
| Pancreas | 1 (2.4%) |
| Penile | 1 (2.4%) |
| Peritoneum | 1 (2.4%) |
| Prostate | 1 (2.4%) |
| Renal pelvis | 1 (2.4%) |
| Unknown | 1 (2.4%) |
| **Male (n=18)** | |
| Lung | 4 (22.2%) |
| Colon | 2 (11.1%) |
| Gastric | 2 (11.1%) |
| Bladder | 1 (5.6%) |
| Duodenum | 1 (5.6%) |
| Extramammary Paget’s | 1 (5.6%) |
| Gum | 1 (5.6%) |
| Hepatic | 1 (5.6%) |
| Lymphoma | 1 (5.6%) |
| Oropharynx | 1 (5.6%) |
| Penile | 1 (5.6%) |
| Prostate | 1 (5.6%) |
| Thyroid | 1 (5.6%) |
| **Female (n=24)** | |
| Breast | 4 (16.7%) |
| Gastric | 3 (12.5%) |
| Bowen disease | 2 (8.3%) |
| Cervix | 2 (8.3%) |
| Colon | 2 (8.3%) |
| Oropharynx | 2 (8.3%) |
| Thyroid | 2 (8.3%) |
| Gallbladder | 1 (4.2%) |
| Oral | 1 (4.2%) |
| Ovarian | 1 (4.2%) |
| Pancreas | 1 (4.2%) |
| Peritoneum | 1 (4.2%) |
| Renal pelvis | 1 (4.2%) |
| Unknown | 1 (4.2%) |
| Percentages are within-section percentages. | |
| Rows are ranked independently within Overall, Male, and Female. | |

Legend:

Supplementary Table S2. Types of malignancy among patients with cancer-associated myositis. Data are presented as n (%). Percentages are calculated within each section (overall, male, and female). Each patient contributed one malignancy diagnosis within the cancer-associated myositis window.
