# Supplementary material for: A five-factor risk-stratification model for cancer-associated myositis in idiopathic inflammatory myopathies and stage-specific survival analysis: a multicentre Japanese MYKO cohort study
Source: Front Immunol. 2026 Aug 11;17:1914962. doi: 10.3389/fimmu.2026.1914962 (PMC13504253; doi:10.3389/fimmu.2026.1914962)
Supplement: Supplementary file 3 [file Table3.docx]

**Supplementary Table S3. All logistic regression model combinations for cancer-associated myositis prediction**

| Predictors | k | AUC | Sensitivity | Specificity | AIC |
| --- | --- | --- | --- | --- | --- |
| TIF1γ + CRP ≥ 0.17 mg/dL + DM + Age ≥ 69 years + Family history | 5 | 0.857 | 92.5 | 65.1 | 187.3 |
| TIF1γ + CRP ≥ 0.17 mg/dL + Age ≥ 69 years + Family history | 4 | 0.851 | 92.5 | 65.5 | 186.2 |
| TIF1γ + DM + Age ≥ 69 years + Family history | 4 | 0.839 | 77.5 | 75.3 | 199.8 |
| TIF1γ + Age ≥ 69 years + Family history | 3 | 0.831 | 65.0 | 82.9 | 199.4 |
| TIF1γ + CRP ≥ 0.17 mg/dL + DM + Family history | 4 | 0.809 | 80.0 | 73.7 | 199.6 |
| TIF1γ + CRP ≥ 0.17 mg/dL + Family history | 3 | 0.807 | 80.0 | 73.7 | 197.8 |
| CRP ≥ 0.17 mg/dL + DM + Age ≥ 69 years + Family history | 4 | 0.801 | 75.0 | 71.1 | 215.6 |
| TIF1γ + CRP ≥ 0.17 mg/dL + DM + Age ≥ 69 years | 4 | 0.794 | 57.5 | 87.5 | 195.0 |
| TIF1γ + CRP ≥ 0.17 mg/dL + Age ≥ 69 years | 3 | 0.789 | 57.5 | 89.1 | 193.9 |
| TIF1γ + DM + Age ≥ 69 years | 3 | 0.782 | 65.0 | 82.9 | 204.4 |
| CRP ≥ 0.17 mg/dL + Age ≥ 69 years + Family history | 3 | 0.779 | 80.0 | 68.1 | 220.4 |
| TIF1γ + DM + Family history | 3 | 0.773 | 85.0 | 56.2 | 214.3 |
| CRP ≥ 0.17 mg/dL + DM + Age ≥ 69 years | 3 | 0.771 | 77.5 | 65.8 | 221.0 |
| TIF1γ + Family history | 2 | 0.767 | 85.0 | 56.2 | 212.8 |
| DM + Age ≥ 69 years + Family history | 3 | 0.762 | 65.0 | 77.3 | 224.6 |
| TIF1γ + Age ≥ 69 years | 2 | 0.759 | 65.0 | 82.9 | 204.0 |
| CRP ≥ 0.17 mg/dL + Age ≥ 69 years | 2 | 0.739 | 97.5 | 34.5 | 225.3 |
| CRP ≥ 0.17 mg/dL + DM + Family history | 3 | 0.734 | 75.0 | 61.8 | 231.4 |
| TIF1γ + CRP ≥ 0.17 mg/dL + DM | 3 | 0.730 | 32.5 | 97.7 | 208.3 |
| Age ≥ 69 years + Family history | 2 | 0.728 | 75.0 | 61.5 | 230.6 |
| TIF1γ + CRP ≥ 0.17 mg/dL | 2 | 0.725 | 32.5 | 97.0 | 206.5 |
| DM + Age ≥ 69 years | 2 | 0.725 | 80.0 | 55.6 | 227.7 |
| CRP ≥ 0.17 mg/dL + Family history | 2 | 0.711 | 57.5 | 76.3 | 233.7 |
| CRP ≥ 0.17 mg/dL + DM | 2 | 0.681 | 87.5 | 38.5 | 237.1 |
| TIF1γ + DM | 2 | 0.663 | 32.5 | 97.0 | 220.6 |
| DM + Family history | 2 | 0.661 | 85.0 | 38.5 | 241.7 |
| Age ≥ 69 years | 1 | 0.653 | 45.0 | 85.5 | 233.2 |
| TIF1γ | 1 | 0.648 | 32.5 | 97.0 | 219.3 |
| CRP ≥ 0.17 mg/dL | 1 | 0.630 | 87.5 | 38.5 | 239.2 |
| Family history | 1 | 0.626 | 65.0 | 57.9 | 245.1 |
| DM | 1 | 0.595 | 52.5 | 66.4 | 246.0 |
| Models were ranked by AUC, then AIC. | | | | | |
| AUC was calculated from ROC curves of predicted probabilities from each logistic regression model. | | | | | |
| Sensitivity and specificity are reported as percentages and were calculated at the optimal probability threshold based on the Youden index. | | | | | |
| Abbreviations: AUC, area under the receiver operating characteristic curve; AIC, Akaike information criterion; CRP, C-reactive protein; DM, dermatomyositis. | | | | | |

Legend:

Supplementary Table S3. All logistic regression model combinations for prediction of cancer-associated myositis. All 31 possible combinations of the candidate predictors were evaluated. Models were ranked by area under the receiver operating characteristic curve and then by Akaike information criterion. Sensitivity and specificity are presented as percentages and were calculated at the optimal probability threshold determined by the Youden index for each model. k denotes the number of predictors included in each model.
