# Supplementary material for: A five-factor risk-stratification model for cancer-associated myositis in idiopathic inflammatory myopathies and stage-specific survival analysis: a multicentre Japanese MYKO cohort study
Source: Front Immunol. 2026 Aug 11;17:1914962. doi: 10.3389/fimmu.2026.1914962 (PMC13504253; doi:10.3389/fimmu.2026.1914962)
Supplement: Supplementary file 4 [file Table4.docx]

Supplementary Table S4. Observed and model-predicted probability of cancer-associated myositis according to the number of final-model risk factors present

| Number of final-model risk factors present | Number of patients | Number of CAM cases | Observed CAM frequency, % | Model-predicted probability of CAM, median [IQR], % | Model-predicted probability of CAM, range, % |
| --- | --- | --- | --- | --- | --- |
| 0 | 51 | 0 | 0.0 | 0.5 [0.5–0.5] | 0.5–1.2 |
| 1 | 117 | 5 | 4.3 | 3.2 [2.1–3.2] | 0.7–11.4 |
| 2 | 118 | 12 | 10.2 | 11.2 [4.8–13.0] | 3.1–47.3 |
| 3 | 49 | 15 | 30.6 | 20.5 [18.3–43.5] | 14.2–92.1 |
| ≥4 | 9 | 8 | 88.9 | 85.8 [81.7–87.4] | 53.6–96.9 |
| This descriptive analysis included 344 patients with complete data for all five final-model risk factors. | | | | | |
| Risk-factor count was used only as a descriptive clinical summary and not as an unweighted scoring system. | | | | | |
| Model-predicted probabilities were calculated using the final five-factor logistic regression model. | | | | | |
| Because the five factors do not contribute equally to CAM risk, predicted probabilities may vary within the same risk-factor-count category. | | | | | |
| Patients with four or five risk factors were combined into a ≥4 category because only one patient had all five risk factors. | | | | | |
| Abbreviations: CAM, cancer-associated myositis; IQR, interquartile range. | | | | | |

Legend：

This descriptive analysis included 344 patients with complete data for all five final-model risk factors. Risk-factor count was used only as a descriptive clinical summary and not as an unweighted scoring system. Model-predicted probabilities were calculated using the final five-factor logistic regression model. Because the five factors contributed unequally to CAM risk, predicted probabilities varied within the same risk-factor-count category. Patients with four or five risk factors were combined into the ≥4 category because only one patient had all five risk factors.
**Abbreviations:** CAM, cancer-associated myositis; IQR, interquartile range.
